# Autogenous Transplantation of Teeth Across Clinical Indications: A Systematic Review and Meta-Analysis

**DOI:** 10.3390/jcm14145126

**Published:** 2025-07-18

**Authors:** Martin Baxmann, Karin Christine Huth, Krisztina Kárpáti, Zoltán Baráth

**Affiliations:** 1 Department of Orthodontics, Faculty of Education and Research, DTMD University, 9516 Luxembourg, Luxembourg; 2 Department of Prosthodontics, Faculty of Dentistry, University of Szeged, 6720 Szeged, Hungary; barath.zoltan@stoma.szote.u-szeged.hu; 3 Department of Conservative Dentistry, Periodontology and Digital Dentistry, LMU University Hospital, LMU Munich, 80336 Munich, Germany; karin.huth@med.uni-muenchen.de; 4 Department of Orthodontics and Pediatric Dentistry, Faculty of Dentistry, University of Szeged, 6720 Szeged, Hungary; karpati.krisztina@stoma.szote.u-szeged.hu

**Keywords:** transalveolar transplantation, autotransplantation, tooth survival, orthodontic treatment, orthodontic planning

## Abstract

Autogenous tooth transplantation offers a biologically favorable approach to tooth replacement, preserving the periodontal ligament, promoting alveolar development, and maintaining proprioception. **Background/Objectives**: Its broader clinical applicability is limited by variability in techniques and outcome definitions. This systematic review and meta-analysis evaluated the clinical success of autogenous tooth transplantation across donor tooth types, developmental stages, surgical techniques, and fixation methods. **Methods**: Following PRISMA 2020 guidelines and a PROSPERO-registered protocol (CRD42024625550), five databases and the gray literature were searched through July 2025. Eligible studies reported clinical outcomes for autogenous tooth transplantation. Risk of bias was assessed using the Newcastle–Ottawa Scale. A random-effects meta-analysis of logit-transformed proportions was conducted, with subgroup and sensitivity analyses by tooth type, root development stage, surgical technique, and fixation method. **Results**: Twenty studies involving 1366 transplanted teeth were included. The pooled success rate was 94.0% (95% CI: 22.5–99.9%) across follow-up periods ranging from one month to twenty-nine years. However, interpretation is limited by distinct heterogeneity (I^2^ = 99.8%) and the wide confidence interval. Subgroup analyses by tooth type, root maturity, surgical technique, and fixation method are, therefore, emphasized to support clinical interpretation. **Conclusions**: Autogenous tooth transplantation achieves consistently high success across clinical contexts when biologic handling is respected. These findings support its broader use in dental and orthodontic practice and underscore the need for standardized outcome reporting and prospective research. Interpretation is limited by heterogeneity and variation in reporting standards.

## 1. Introduction

Autogenous tooth transplantation (ATT)—the surgical repositioning of a donor tooth within the same individual—has been explored for decades as an alternative to prosthetic replacement, particularly in adolescents and young adults [1]. When indicated, the procedure allows for the preservation of alveolar bone, maintenance of proprioception, and long-term biological integration without the constraints of implant-based rehabilitation [2,3,4]. In growing patients, autotransplantation offers distinct advantages over fixed prostheses and implants, which are contraindicated until skeletal maturity [5]. Autotransplantation has shown clinical utility in cases of traumatic tooth loss, particularly in pediatric patients, where preserving alveolar development and delaying prosthetic intervention is advantageous [6]. Specific protocols, such as two-phase autotransplantation—in which primary teeth are transplanted first, followed by permanent teeth—have been introduced to support anterior rehabilitation in young patients after traumatic tooth loss [7]. Transplantation following dental trauma has also been associated with favorable esthetic and functional outcomes in select cases [8].

As clinical applications have expanded, so too have refinements in surgical technique and donor selection. Recent clinical guidelines and experimental models have shifted focus toward biologically conservative approaches that emphasize preservation of the periodontal ligament (PDL), minimization of extraoral time, and atraumatic extraction techniques [5,9]. These biologic priorities have become central to case planning and are increasingly considered more influential than traditional parameters such as donor tooth morphology or anchorage suitability. Moreover, autotransplantation, intentional replantation, and surgical extrusion are now often discussed under a shared biologic and procedural framework due to their overlapping reliance on PDL vitality and wound healing principles [10].

Although prior reviews have addressed selected aspects of autotransplantation, such as maxillary canine transplants, orthodontic applications, or third molars, no systematic review to date has comprehensively evaluated clinical outcomes across donor tooth types, recipient sites, and surgical protocols. This review expands the scope by including all clinical indications, developmental stages, and fixation strategies, thereby offering a broader synthesis that enables comparison across heterogeneous clinical contexts. Moreover, limited attention has been paid to the influence of developmental stage, donor tooth type, or transplantation indication on long-term prognosis. As a result, clinicians lack a consolidated evidence base to guide patient selection, surgical planning, and follow-up strategies across diverse clinical contexts.

This systematic review and meta-analysis was conducted to evaluate the clinical outcomes of autogenous tooth transplantation across a wide range of surgical approaches, donor tooth types, developmental stages, and treatment indications. Specifically, this review addresses ongoing controversies regarding the prognosis of mature versus immature teeth, the comparative effectiveness of various stabilization techniques, and the role of biologic adjuncts such as platelet-rich fibrin (PRF). Studies published from database inception through July 2025 were included to capture both historical and contemporary data, allowing for the evaluation of long-term outcomes and technique evolution. Guided by a structured PICO framework, the review focused on tooth survival, root resorption, ankylosis, and pulp vitality as primary outcomes. Secondary aims included identifying factors associated with success or failure, such as surgical technique, stabilization method, and root development status, as well as examining orthodontic and restorative considerations in treatment planning. The review sought to synthesize both quantitative and qualitative evidence to inform clinical practice, support procedural decision-making, and identify priorities for future research.

## 2. Materials and Methods

### 2.1. Protocol and Registration

**Figure 1:**
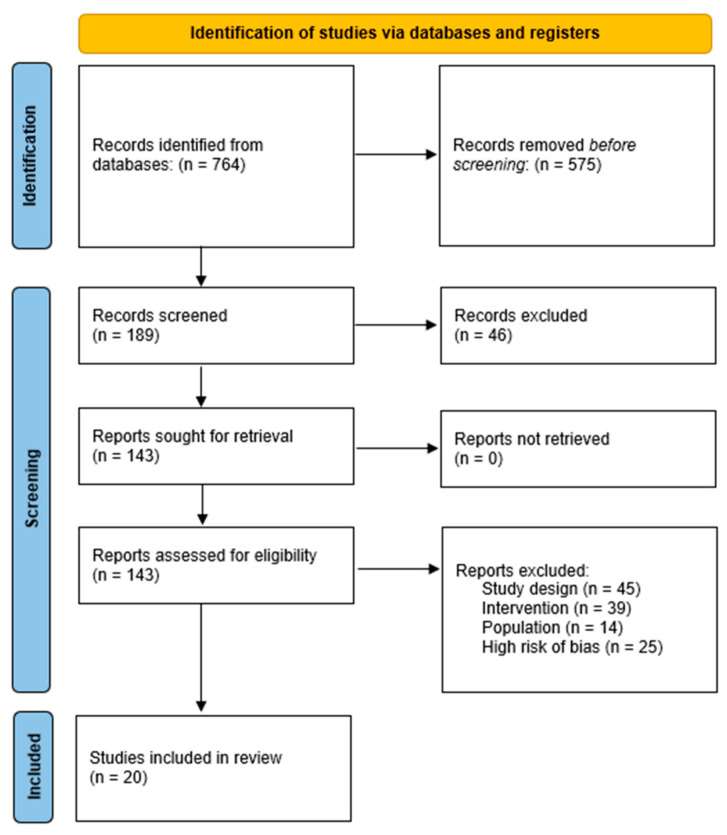
PRISMA Flow Diagram.

This systematic review was conducted and reported in accordance with the PRISMA 2020 guidelines. The study selection process is illustrated in a PRISMA 2020 flow diagram (Figure 1). The protocol was registered with PROSPERO (CRD42024625550). Several minor revisions were made to the original PROSPERO-registered protocol to reflect the expanded scope of the review. Specifically, eligibility criteria were broadened to include all donor tooth types, recipient sites, and surgical techniques, rather than limiting the review to molar transplants as initially planned. These changes were documented in the PROSPERO registration (CRD42024625550), which was updated accordingly to maintain methodological transparency. A completed PRISMA checklist has been submitted as a supplementary file.

### 2.2. Eligibility Criteria

Eligible studies included human participants of any age who had undergone autogenous transplantation of a permanent or primary tooth. All surgical approaches were considered, including the transplantation of erupted or impacted teeth, extraoral preparation, and placement into natural or surgically prepared sockets. Studies with or without comparison groups were eligible for inclusion, provided they reported at least one relevant clinical outcome such as tooth survival, root resorption, ankylosis, or pulp vitality.

Eligible studies were required to report at least one clinically relevant outcome such as tooth survival, root resorption, ankylosis, or pulp vitality. Studies that used vague or undefined criteria for success (e.g., referring only to “successful transplantation” without clinical or radiographic detail) were excluded during full-text screening. Among the included studies, reported success definitions varied and were retained as originally described. To facilitate synthesis, outcome data were later grouped into thematic categories based on shared clinical constructs. A summary of these definitions is provided in Appendix C to support interpretability of pooled and stratified analyses.

Secondary outcomes included orthodontic planning considerations, criteria for clinical success, and complications or causes of failure. Studies were excluded if they did not present original clinical data, involved animal or in vitro models, or were not available in full text despite reasonable retrieval efforts. Conference abstracts without full publications, reviews, editorials, and commentaries were also excluded. No restrictions were applied based on language, publication year, or geographic location.

### 2.3. Search Strategy

A comprehensive electronic search of PubMed/MEDLINE, Embase, the Cochrane Library, Scopus, and Web of Science was conducted from inception to July 2025. The search strategy combined free-text keywords and controlled vocabulary terms related to autogenous tooth transplantation, including terms such as “autotransplantation”, “transalveolar transplantation”, “canine transplantation”, “success rate”, and “root resorption”. Boolean operators, truncation, and wildcards were applied to optimize retrieval. Reference lists of the included studies and relevant reviews were manually screened to identify additional eligible articles. The gray literature was not excluded a priori and was considered during the database and manual searches. However, no eligible gray literature records were identified during the screening process. To ensure completeness, the Consensus.ai platform was used to cross-check potentially overlooked studies.

### 2.4. Study Selection

All search results were imported into Covidence (version released May 2022; Covidence.org, Melbourne, Australia) for deduplication and screening. Two reviewers independently screened titles and abstracts to identify potentially eligible studies, followed by full-text assessment of selected articles. Disagreements at either stage were resolved through discussion or adjudication by a third reviewer. The study selection process was documented using a PRISMA flow diagram, detailing the number of records identified, screened, excluded, and included.

### 2.5. Data Extraction

Data were extracted independently by two reviewers using a standardized template. Extracted variables included study design, sample size, participant demographics, donor tooth type and developmental stage, surgical technique, recipient socket preparation, stabilization method and duration, follow-up period, and reported outcomes. Any discrepancies in extraction were resolved through consensus. When necessary, study authors were contacted for clarification or missing data. Success was defined according to each study’s reported criteria and typically included survival of the transplanted tooth, absence of clinical symptoms or radiographic pathology, and in some cases, evidence of root development or functional integration. In studies that reported more than one treatment group—such as those stratified by root development stage, fixation method, or surgical technique—each group was extracted as a distinct study arm, provided that the groups reported separate sample sizes and outcome data. This allowed for the inclusion of multiple non-overlapping effect sizes from the same publication, which were analyzed independently in the quantitative synthesis to support subgroup comparisons.

### 2.6. Risk of Bias Assessment

Risk of bias was assessed independently by two reviewers using tools appropriate to the study design. Observational studies were evaluated using the ROBINS-I tool, and case series or case reports were assessed using a modified version of the Joanna Briggs Institute (JBI) checklist. Each study was categorized as having low, moderate, or serious risk of bias based on predefined criteria. Discrepancies were resolved through discussion, and if consensus could not be reached, a third reviewer was consulted. A formal GRADE assessment was not performed due to the observational and non-comparative nature of the included studies, along with the absence of direct comparisons or effect estimates required for standard GRADE criteria. However, overall confidence in the evidence was narratively evaluated in terms of study design, risk of bias, consistency of directionality, and precision of estimates, as discussed in the limitations.

### 2.7. Data Synthesis and Analysis

Quantitative synthesis was performed using R version 4.4.2, employing the metafor and meta packages. Proportions were logit-transformed and pooled using a random-effects model to account for heterogeneity. Between-study variability was evaluated using the I^2^ statistic, tau^2^, and Cochran’s Q test. Subgroup analyses were conducted to examine potential differences in outcomes based on donor tooth type, developmental stage, surgical technique, stabilization method, recipient site, and clinical indication. Sensitivity analyses excluded studies with serious risk of bias to assess the robustness of the findings. In addition to the quantitative meta-analysis, qualitative findings related to outcome definitions, orthodontic planning, and patterns of failure were synthesized thematically to contextualize clinical implications. Publication bias was evaluated through visual inspection of funnel plots and tested statistically using Egger’s regression test for funnel plot asymmetry. To promote transparency and reproducibility, the R script used for data synthesis and the standardized data extraction form have been made available as supplementary material (Supplementary Files S1 and S2). The R code includes preprocessing steps, meta-analysis commands, and plotting functions used to generate the forest and funnel plots. Any patient-level data were not shared as none were used in this aggregate analysis.

Subgroup analyses were pre-specified in the PROSPERO-registered protocol to explore potential sources of heterogeneity based on donor tooth type, root development stage, surgical technique, fixation method, recipient site, and clinical indication. Meta-regression was considered but ultimately not performed due to limited covariate reporting across studies and insufficient subgroup sizes to yield reliable model estimates. These limitations constrained the ability to statistically model heterogeneity beyond subgroup comparisons.

In addition to the quantitative meta-analysis, qualitative findings—particularly those related to outcome definitions, orthodontic considerations, and patterns of failure—were analyzed using a narrative synthesis approach. Thematic patterns were identified manually by two reviewers, who independently extracted descriptive elements from eligible studies. Discrepancies were resolved through discussion to ensure reliability of interpretation. To support interpretability, the underlying assumptions of the statistical models are as follows: The random-effects model used in this review assumes that the true effects vary across studies and are normally distributed. This approach accounts for both within-study sampling errors and between-study heterogeneity through the estimation of a between-study variance component (τ^2^). Unlike fixed-effects models, it does not assume homogeneity of variances across studies. The logit transformation was applied to stabilize variance and improve normality in the distribution of proportions, particularly in studies with extreme event rates.

## 3. Results

A total of 764 records were identified through database searches. After removing 575 duplicates and irrelevant records prior to screening, 189 records remained. Of these, 46 were excluded during title and abstract screening. The remaining 143 full-text reports were assessed for eligibility. Among these, 45 were excluded due to study design, 39 due to intervention criteria, 14 based on population mismatch, and 25 due to high risk of bias. Ultimately, 20 studies met inclusion criteria and were included in the systematic review (Figure 1).

Because some studies included more than one treatment group based on root development stage or intervention type, a total of 29 study arms were included in the quantitative synthesis. Sample sizes per group ranged from 1 to 182 transplanted teeth. Most arms involved permanent teeth (n = 28); only one group reported on primary teeth. Root development was classified as immature (n = 10), mature (n = 11), or mixed (n = 8). Third molars were the most commonly transplanted tooth type, followed by premolars, canines, and molars. Surgical approaches included standard (n = 10), modified (n = 9), immediate (n = 4), biologic-assisted (n = 1), and 3D-guided techniques (n = 2) (See Appendix A for approach definitions). Fixation methods varied and included suture only (n = 5), splinting (n = 6), suture or splint (n = 6), adhesive fixation (n = 4), and minimal or no fixation (n = 1); the remainder were not specified. Reported follow-up durations ranged from 1 month to 29 years, and success rates ranged from 60.0% to 100.0%. A full summary of study characteristics, methodological details, and reported outcomes is provided in Supplementary File S3.

Risk of bias was assessed using the Newcastle–Ottawa Scale or an adapted version. Of the 20 included studies, 10 were rated as having a low risk of bias and 10 as moderate (Table 1). No study was classified as high risk, as studies meeting inclusion criteria with a high overall risk of bias had been excluded. A list of excluded studies due to high risk of bias can be viewed in the Appendix B. Funding sources for each included study were reviewed. Most studies did not report funding sources; however, when available, this information is provided in Supplementary File S4.

Twenty studies including 1366 transplanted teeth were included in the meta-analysis. The random-effects model yielded a pooled logit-transformed success rate of 2.81 (95% CI: −1.24 to 6.86), corresponding to an estimated success proportion of 94.3% (95% CI: 22.5% to 99.9%). Between-study heterogeneity was high, with I^2^ = 99.75%, τ^2^ = 72.56, and Cochran’s Q = 10,708.60 (df = 27, *p* < 0.001). Given the wide confidence interval and high heterogeneity, further subgroup analyses were conducted to explore potential sources of variability, including donor tooth type, developmental stage, surgical technique, fixation method, and recipient site.

**Figure 2:**
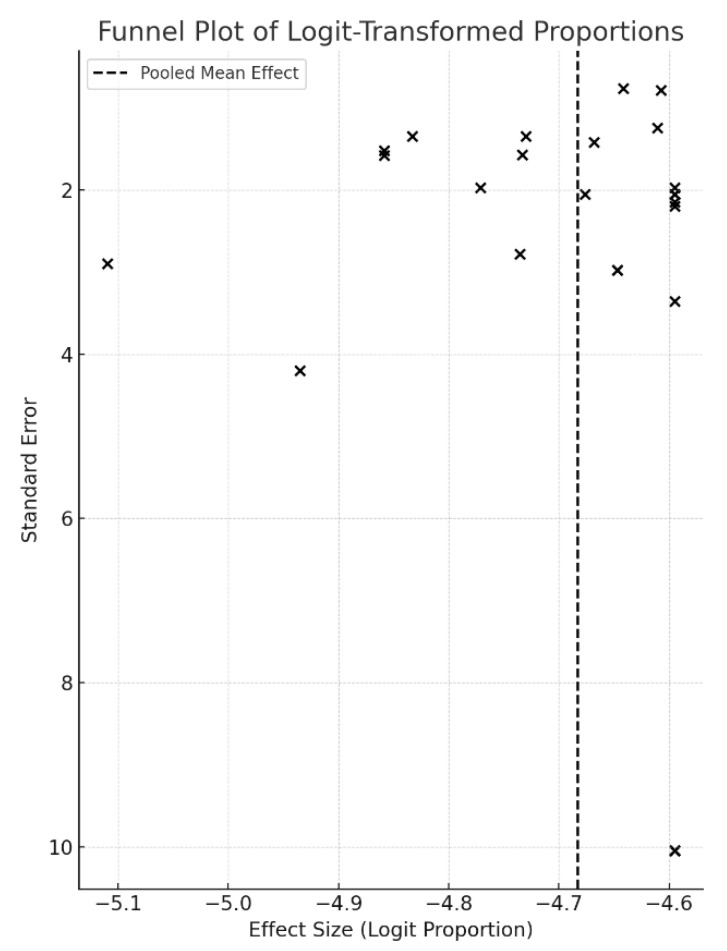
Funnel Plot of Logit-Transformed Survival Proportions. Note: Each point represents an included study. The vertical dashed line indicates the pooled mean effect. Asymmetry in the plot suggests potential publication bias, supported by Egger’s test (*p* < 0.001).

Publication bias was assessed using a funnel plot and Egger’s regression test. Visual inspection of the funnel plot suggested asymmetry, and Egger’s test confirmed significant small-study effects (*p* < 0.001), indicating potential publication bias in the pooled survival estimates (Figure 2).

When subgroup analyses were stratified by tooth type, the pooled success rate was 98.0% (95% CI: 95.9% to 99.0%) for premolars, 92.9% (95% CI: 87.2% to 96.2%) for molars, 89.4% (95% CI: 79.4% to 95.0%) for third molars, and 93.5% (95% CI: 89.2% to 96.2%) for canines. Heterogeneity across tooth type subgroups ranged from I^2^ = 84.0% to 96.1%. By dentition, permanent teeth demonstrated a pooled success rate of 95.0% (95% CI: 90.5% to 97.6%; I^2^ = 96.5%). Only one study evaluated primary teeth, and a pooled estimate was not calculated for this group.

When grouped by root development stage, the pooled success rate for immature teeth was 96.8% (95% CI: 93.1% to 98.7%; I^2^ = 92.4%), for mature teeth 89.3% (95% CI: 78.6% to 95.1%; I^2^ = 94.6%), and for mixed-stage teeth 90.6% (95% CI: 84.7% to 94.5%; I^2^ = 89.9%). Subgroup analysis by surgical technique showed pooled success rates of 94.9% (95% CI: 90.5% to 97.4%; I^2^ = 91.0%) for standard autotransplantation, 90.6% (95% CI: 84.5% to 94.6%; I^2^ = 93.5%) for modified techniques (e.g., socket reshaping, donor root contouring, alternative fixation methods), 93.0% (95% CI: 80.5% to 97.8%; I^2^ = 89.3%) for immediate autotransplantation (i.e., placement into a freshly extracted socket during the same surgical session), and 96.6% (95% CI: 84.3% to 99.4%; I^2^ = 86.8%) for 3D-guided procedures (See Appendix A for definitions). Studies employing biologic or graft-enhanced techniques reported a pooled success rate of 100.0%, with no observed heterogeneity (I^2^ = 0%). However, this estimate was based on a very small number of study arms (n = 2), and the narrow group size limits generalizability despite the use of a random-effects model.

Fixation methods also showed variation in success rates (See Appendix A for definition). Pooled estimates were 95.4% (95% CI: 91.7% to 97.5%; I^2^ = 88.2%) for suture-only techniques (e.g., transgingival sutures without rigid stabilization), 93.3% (95% CI: 87.2% to 96.6%; I^2^ = 89.9%) for wire or resin splints (e.g., passive splinting using orthodontic wire or resin composite), 91.6% (95% CI: 84.3% to 95.7%; I^2^ = 91.9%) for combined suture or splint approaches (where the method varied across patients or was not isolated), and 92.8% (95% CI: 85.2% to 96.7%; I^2^ = 88.5%) for adhesive fixation (typically resin-based bonding between the donor and adjacent teeth). Studies with no or minimal fixation reported a pooled success rate of 100.0%, with I^2^ = 0%. However, this estimate is based on a small number of study arms with limited sample sizes and should not be interpreted as evidence that fixation is unnecessary.

Recipient site classification also revealed variation in pooled success rates. When stratified by anatomical donor region, pooled success was 98.2% (95% CI: 94.3% to 99.5%) for anterior sites and 92.6% (95% CI: 89.2% to 94.9%) for posterior sites. A mandibular vs. maxillary comparison showed pooled rates of 91.8% (95% CI: 88.3% to 94.4%) for mandibular transplants and 91.6% (95% CI: 88.1% to 94.2%) for maxillary transplants. When grouped by recipient tooth category, incisor/canine sites had a pooled success rate of 96.6% (95% CI: 91.7% to 98.7%), while premolar/molar sites had a pooled rate of 92.7% (95% CI: 89.5% to 95.0%). While these findings suggest marginally higher success in anterior and mandibular regions, subgroup sizes varied, and recipient site information was inconsistently reported in several studies. In some cases, classification was ambiguous or included multiple locations within the same cohort, limiting the precision of anatomical comparisons. Accordingly, recipient site analyses should be interpreted as general trends rather than discrete or mutually exclusive categories.

Reporting of adverse outcomes, such as root resorption, ankylosis, and pulp necrosis, varied across the included studies. While a small number of studies provided detailed data on these events, the majority either did not report them explicitly or only referenced them narratively. Among those that did report adverse events, the overall incidence of root resorption ranged from 0% to 13%, with higher rates generally observed in mature teeth or in cases lacking biologic handling protocols. Ankylosis was infrequently reported and typically limited to cases involving extended extraoral time or inadequate stabilization. Due to this variability in definitions and incomplete reporting, pooled analyses of these complications were not performed. However, studies emphasizing atraumatic technique, short extraoral duration, and biologic adjuncts tended to report fewer adverse outcomes.

## 4. Discussion

This systematic review and meta-analysis synthesized 29 arms across 20 studies involving a total of 1366 transplanted teeth. Success rates were directionally favorable across most subgroups, with outcomes ranging from 89% to 98% depending on donor tooth type, root development stage, and surgical protocol. However, substantial statistical heterogeneity (I^2^ > 90%) was present in nearly all pooled analyses, limiting the interpretability of these proportions as precise estimates of effect. The inclusion of one study involving primary dentition also introduced variation in outcome definitions and procedural details [16]. Some numerical trends—such as higher survival among premolars or immature teeth—did not achieve statistical significance. Nevertheless, the consistently high survival rates across diverse clinical contexts reinforce the clinical viability of autotransplantation when biologic principles are respected.

Root maturity played a notable role in reported outcomes. Immature roots were generally associated with continued development and high success rates, whereas mature teeth also demonstrated favorable outcomes when biologically respectful handling was observed. Two studies found that early or immediate root canal therapy (RCT) in mature transplants led to fewer failures, particularly when endodontic intervention was not delayed beyond the first two weeks postoperatively [13,15]. A third study reported consistently high survival when RCT was performed immediately after transplantation in all mature donor teeth [17]. In contrast, several other studies either lacked mature donor teeth, failed to specify RCT protocols, or omitted relevant timing details, limiting further analysis. The reported benefit of timely RCT in mature teeth warrants additional prospective investigation.

The surgical technique appeared to significantly influence outcomes, often independent of donor tooth type or root maturity. Multiple studies suggested that atraumatic extraction, precise adaptation of the donor root to the recipient socket, and minimization of extraoral time were key determinants of success, particularly in mature teeth [11,20]. The absence of root resorption or vitality loss in many mature transplants underscores the importance of biologically respectful technique, reinforcing that successful outcomes can be achieved even the case of apical closure [17].

Donor tooth selection varied across studies, with third molars being most common, followed by premolars and canines. While third molars showed slightly lower survival in some reports, they still achieved high success overall. Subgroup analysis also indicated slightly higher pooled success rates in anterior and mandibular recipient regions compared to posterior and maxillary sites [16,21,22,27]. This may reflect biomechanical differences in occlusal loading or ease of surgical access. Some studies involving only posterior sites also reported favorable outcomes when structured protocols and biologic adjuncts were used [12,16,17,22,23,27,29]. In pediatric cases, even primary tooth transplantation yielded functional and esthetic benefits, despite lower reported survival [16].

Biologic adjuncts were applied in a small number of studies and included platelet-rich fibrin (PRF), concentrated growth factor (CGF), and partially demineralized dentin matrix. These approaches aimed to enhance healing, particularly in compromised sockets, and were generally associated with favorable outcomes [11,12,30]. However, these techniques were not compared directly with standard protocols, and data remain limited. Similarly, 3D-guided autotransplantation was reported to improve placement precision and reduce extraoral time, but did not show a significant improvement in pooled survival rates [27,30].

Fixation techniques varied and included splints, sutures, adhesive fixation, or no stabilization at all. Despite these differences, no specific fixation method consistently outperformed others in terms of success. Studies emphasized that atraumatic handling, close adaptation of the donor root to the socket, and rapid replantation were more important than the specific type of fixation [18,19,22]. Radiographic evidence of early bone remodeling within three months post-transplant supported the notion that biological healing processes are robust when key procedural principles are followed [30]. Long-term follow-up further confirmed that outcomes were stable over time when biologically respectful technique was maintained [16,20].

Two-stage surgical approaches, in which the recipient site was allowed to heal before transplantation, were associated with improved outcomes in mature teeth. These techniques reportedly reduced the incidence of root resorption and improved long-term retention [24,28]. Adjunctive procedures such as socket grafting or root surface conditioning also contributed to favorable results in selected cases [23,25]. A unifying theme across these varied approaches was the importance of preserving periodontal ligament vitality and promoting early functional integration.

Operator skill emerged as potential confounder across studies. Those involving a single experienced operator and standardized protocols generally reported more consistent success, while retrospective studies or those involving multiple surgeons tended to show greater outcome variability. Unfortunately, reporting on surgeon experience, intraoperative techniques, and procedural consistency was often incomplete, preventing a more granular analysis of these influences.

Despite the high statistical heterogeneity, no individual subgroup exhibited contradictory results. The variability observed likely reflects differences in surgical protocols, follow-up duration, case selection, and success definitions rather than fundamental disagreement on clinical effectiveness. Appendix C summarizes the diverse success criteria used across studies. Although a general definition of success was adopted for synthesis purposes, readers are cautioned to interpret pooled estimates as indicative of broad clinical trends, not as definitive effect sizes.

### 4.1. Clinical Implications

The findings of this review support the clinical viability of ATT across a range of donor tooth types, recipient sites, and developmental stages. When performed with biologically respectful technique, ATT can achieve high success rates and serve as a valuable alternative to dental implants or prostheses, particularly in growing patients. Clinical scenarios where ATT may be especially beneficial include space maintenance following traumatic tooth loss or agenesis, alveolar bone preservation during development, deferral of prosthetic replacement in adolescents, and cases where orthodontic space closure is either not feasible or contraindicated.

The pooled success rates were highest for immature teeth and premolars, suggesting that these donor types may be particularly favorable when available. For mature donor teeth, timely root canal therapy is suggested to reduce the risk of pulp necrosis and resorption. Use of atraumatic extraction, reduced extraoral handling time, and stable but non-rigid fixation methods appear to enhance outcomes. Furthermore, the consistent performance of both anterior and posterior recipient sites—despite heterogeneity in reporting—supports case-by-case planning rather than rigid anatomical exclusion. These findings encourage a broader clinical adoption of ATT when biologic feasibility is present and case selection is thoughtful.

### 4.2. Future Directions

While this review demonstrates consistently high success rates for ATT, several areas warrant further investigation to strengthen clinical guidance and standardization. First, prospective studies comparing fixation methods (e.g., suture, splinting, and no fixation) would help clarify their relative impact on outcomes. Second, controlled trials examining the use of biologic adjuncts such as platelet-rich fibrin (PRF) could elucidate their role in promoting periodontal healing and revascularization. Third, consensus on success definitions and the development of standardized reporting guidelines for ATT would greatly improve synthesis in future reviews. Finally, studies with longer follow-up durations and greater detail on operator training or surgical expertise could clarify the role of provider skill in procedural success.

### 4.3. Limitations

This review is limited by heterogeneity in study design, wide range of follow-up duration, and outcome definitions. Although pooled success rates were high, methodological differences—such as variation in fixation techniques and differing thresholds for defining “success”—may have influenced outcomes. While a few studies provided insight into the timing and role of root canal therapy, most lacked sufficient detail to evaluate its influence on outcomes systematically. Several included studies were retrospective in design (74%), and only a small number provided long-term follow-up beyond five years. Additionally, publication bias may be present, as unsuccessful cases may be underrepresented in the literature; this was supported by funnel plot asymmetry and a statistically significant Egger’s test (*p* < 0.001). Due to these limitations, findings should be interpreted as indicative of general trends rather than precise effect sizes. Moreover, as is typical in reviews of observational studies, the reliability of extracted data depends on the quality and transparency of reporting in the original studies. Despite the use of formal risk-of-bias tools and the exclusion of high-risk studies in sensitivity analyses, inconsistencies in reporting and variable follow-up durations may affect the precision of pooled outcomes.

## 5. Conclusions

The findings of this review suggest that tooth transplantation is associated with generally favorable outcomes across a range of donor teeth, developmental stages, and surgical protocols. However, substantial heterogeneity across studies and variation in outcome definitions limit the precision and generalizability of pooled estimates. These trends support the clinical promise of autotransplantation while highlighting the need for more standardized, comparative, and long-term research. Regardless of these variations, consistent outcomes were associated with biologically respectful handling and early stabilization of the transplanted tooth through appropriate socket fit and fixation. These results support the reliability of this approach as a treatment modality in contemporary dental and orthodontic practice and underscore the need for further high-quality studies to refine its application.

## Figures and Tables

**Table 1:** Risk of Bias Outcomes.

Study	RoB Tool Used	Overall Risk of Bias
Alkofahi et al., 2020 [11]	Newcastle–Ottawa (adapted)	Moderate
Cahuana-Bartra et al., 2020 [12]	Newcastle–Ottawa (adapted)	Low
Cui et al., 2021 [13]	Newcastle–Ottawa	Moderate
Dixit et al., 2024 [14]	Newcastle–Ottawa	Low
Han et al., 2025 [15]	Newcastle–Ottawa	Low
Hoss et al., 2021 [16]	Newcastle–Ottawa	Moderate
Huth et al., 2013 [17]	Newcastle–Ottawa	Moderate
Keranmu et al., 2021 [18]	Newcastle–Ottawa	Low
Kim et al., 2005 [19]	Newcastle–Ottawa	Moderate
Kimura et al., 2021 [20]	Newcastle–Ottawa	Low
Kvint et al., 2010 [21]	Newcastle–Ottawa	Low
Meinzer et al., 2025 [22]	Newcastle–Ottawa	Low
Murata et al., 2022 [23]	Newcastle–Ottawa (adapted)	Low
Nethander, 1998 [24]	Newcastle–Ottawa (adapted)	Moderate
Nethander et al., 1988 [25]	Newcastle–Ottawa	Low
Nimčenko et al., 2014 [26]	Newcastle–Ottawa (adapted)	Moderate
Park et al., 2022 [27]	Newcastle–Ottawa	Low
Pogrel, 1987 [28]	Newcastle–Ottawa (adapted)	Moderate
Suwanapong et al., 2021 [29]	Newcastle–Ottawa	Moderate
Waikakul et al., 2011 [30]	Newcastle–Ottawa (adapted)	Low

## Data Availability

No new data were created or analyzed in this study.

## Supplementary Materials

The following supporting information can be downloaded at: https://www.mdpi.com/article/10.3390/jcm14145126/s1, reference [31] is cited in Supplementary Materials.

## Abbreviations

The following abbreviations are used in this manuscript:

ATT	Autogenous tooth transplantation
CGF	Concentrated growth factor
PDL	Periodontal ligament
PRF	Platelet-rich fibrin
RCT	Root canal treatment
TPTX	Two-phase transplantation

## Appendix A

**Table A1:** Glossary of Definitions.

Term	Definition
3D-Guided autotransplantation	Use of digital planning and/or 3D-printed surgical guides to facilitate optimal positioning of the donor tooth and minimize extraoral time.
Adhesive fixation	Resin-based bonding directly between the transplanted tooth and adjacent dentition.
Biologic-assisted techniques	Incorporation of regenerative materials such as platelet-rich fibrin (PRF), concentrated growth factor (CGF), or partially demineralized dentin matrix to support healing, particularly in compromised sockets.
Combined suture/splint	Cases where either method was used depending on clinical judgment, or when studies did not report separate outcomes per method.
Immediate autotransplantation	Placement of the donor tooth into a fresh extraction socket during the same surgical session, often following traumatic avulsion or extraction of a non-restorable tooth.
Modified techniques	Procedures that deviate from the standard protocol by incorporating adjustments such as socket reshaping, donor root contouring, atypical fixation methods, or adjustments in surgical sequence to accommodate anatomical or clinical constraints.
No/minimal fixation	Donor tooth placed without active stabilization; relies on socket adaptation and minimal occlusal loading.
Standard autotransplantation	A conventional procedure using biologically respectful handling without adjunctive materials or significant alterations in protocol. Typically involves extraction and placement into a prepared socket without digital planning or additional regenerative strategies.
Suture-only fixation	Transgingival sutures used to secure the donor tooth without the application of rigid splints.
Wire or resin splinting	Use of passive orthodontic wire or composite resin bonded to adjacent teeth to stabilize the transplanted tooth.

## Appendix B

**Table A2:** Studies Omitted Due to High Risk of Bias.

Study (First Author, Year)	Design Type	Overall Risk of Bias
Adamska et al., 2024 [32]	Case report	High
Ahmed Asif et al., 2017 [33]	Case report	High
Asgary and Parhizkar, 2022 [34]	Case report	High
Asgary, 2009 [35]	Case report	High
Asgary, 2023 [36]	Case report	High
Asgary, 2024 [37]	Case report	High
Candeiro et al., 2015 [38]	Case report	High
Chai et al., 2023 [39]	Case report	High
Chaudhary et al., 2015 [40]	Case report	High
Chugh et al., 2012 [41]	Narrative review	High
Ferreira et al., 2015 [42]	Case report	High
Fllippi et al., 1998 [43]	Case report	High
Herrera et al., 2006 [44]	Case report	High
Kamadjaja, 2015 [45]	Case report	High
Kang et al., 2013 [46]	Case reports	High
Kulkarni et al., 2013 [47]	Case report	High
Kumar et al., 2020 [48]	Case report	High
Lee and Kim, 2012 [49]	Case report	High
Marques-Ferreira et al., 2011 [50]	Case report	High
Ravi Kumar et al., 2012 [51]	Case report	High
Reich, 2008 [52]	Case report	High
SamavatiJame et al., 2025 [53]	Case report	High
Teixeira et al., 2006 [54]	Case report	High
Tirali et al., 2013 [55]	Case report	High
Vuletić et al., 2014 [56]	Case report	High

## Appendix C

**Table A3:** Individual Study Definitions of Success.

Study (First Author, Year)	Success Definition
Alkofahi et al. (2020) [11]	Root development
Cahuana-Bartra et al. (2020) [12]	Clinical and radiographic success
Cui et al. (2021) [13]	Clinical and radiographic success
Dixit et al. (2024) [14]	Unspecified/general success
Han et al. (2025) [15]	Clinical and radiographic success
Hoss et al. (2021) [16]	Root development
Huth et al. (2013) [17]	Clinical and radiographic success
Keranmu et al. (2021) [18]	Clinical and radiographic success
Kim et al. (2005) [19]	PDL health/absence of complications
Kimura et al. (2021) [20]	PDL health/absence of complications
Kvint et al. (2010) [21]	Root development
Meinzer et al. (2025) [22]	Initial healing (pain intensity, signs of pathology)
Murata et al. (2022) [23]	PDL health/absence of complications
Nethander (1998) [24]	Retention without pathology
Nethander et al. (1988) [25]	PDL health/absence of complications
Nimčenko et al. (2014) [26]	PDL health/absence of complications
Park et al. (2022) [27]	Retention without pathology
Pogrel (1987) [28]	Clinical and radiographic success
Suwanapong et al. (2021) [29]	Clinical and radiographic success
Waikakul et al. (2011) [30]	PDL health/absence of complications
