# Supplementary material for: Autogenous Transplantation of Teeth Across Clinical Indications: A Systematic Review and Meta-Analysis
Source: J Clin Med. 2025 Jul 18;14(14):5126. doi: 10.3390/jcm14145126 (PMC12295735; doi:10.3390/jcm14145126)
Supplement: Supplementary file 1 [file jcm-14-05126-s001.zip › Supplementary File S2.pdf]

Author_Year	Study_Design	Sample_Size_Teeth	Median_Age	Donor_Tooth_Type	Dentition	Root_Stage	Recipient_Site	Surgical_Technique	Fixation_Method	Success_Definition	Survival_Proportion	Followup_Years	Failure_Proportion	Study_Notes
Alkofahi et	Case repor	1	16	Molar	Permanen	Immature	Not report	Immediate	Not specifi	Root devel	1	2	0	PRF may enhance periodontal healing and root development
Cahuana-B	Case repor	1	16	Premolar	Permanen	Immature	Maxillary c	3D-guided	Suture onl	Clinical an	1	1	0	3D printing minimized extraoral time and improved placement accuracy
Cui et al.	Retrospect	9		Molar	Permanen	Mature	Not report	Standard A	Suture onl	Clinical an	1	0.69	0	RCT improved success
Cui et al.	Retrospect	20		Molar	Permanen	Mature	Not report	Standard A	Suture onl	Clinical an	0.6	0.69	0.4	RCT improved success
Dixit et al.	Prospectiv	12	28	Third mola	Permanen	Mature	Mandibula	Standard A	Not specifi	Unspecifie	0.95	0.5	0.083	Similar success in mature & immature
Dixit et al.	Prospectiv	12	28	Third mola	Permanen	Immature	Mandibula	Standard A	Not specifi	Unspecifie	0.95	0.5	0.083	Similar success in mature & immature
Han et al. (	Retrospect	167	21.5	Third mola	Permanen	Mature	Mandibula	Standard A	Suture or s	Clinical an	0.988	0.55	0.024	RCT led to better outcomes
Hoss et al.	Retrospect	53	9.3	Anterior (c	Primary	Immature	Primary in	Modified A	Suture or s	Root devel	0.77	0.77	0.585	Useful for space & tissue development
Huth et al.	Retrospect	13	12.4	Premolars,	Permanen	Mixed (Im	Not clearly	Standard	Not specifi	Function w	100	3.4	0	Stable outcomes with both mature and immature roots; oral hygiene and surgical care key to success.
Keranmu e	Prospectiv	26	32.4	Third mola	Permanen	Mature	Mandibula	ATT with g	Splint (wir	Clinical an	1	0.5	0	CGF group had 100% success; faster healing, less pain, better initial stability than control
Keranmu e	Prospectiv	26	32.9	Third mola	Permanen	Mature	Mandibula	3D-guided	Not specifi	Clinical an	0.923	0.5	0.077	Control group had lower success and slower healing; CGF beneficial for inflammation and stability
Kim et al. (	Retrospect	182	36	Molar and	Permanen	Mixed	Not report	Standard A	Not specifi	PDL health	0.955	0.67	0.044	<10 min extraoral time correlated with better prognosis
Kimura et	Case repor	1	24	Third mola	Permanen	Mature	Mandibula	Immediate	Adhesive f	PDL health	1	29	0	Demonstrated 29-year long-term success of mature ATT
Kvint et al.	Retrospect	24	15.2	Premolar	Permanen	Immature	Maxillary i	Standard A	Adhesive f	Root devel	1	4.3	0	Premolars transplanted to maxillary incisor region had 100% success; most favorable outcome group
Kvint et al.	Retrospect	71	15.2	Third mola	Permanen	Mixed	Maxillary i	Standard A	Adhesive f	Root devel	0.79	4.3	0.211	Most frequently used donor teeth; 79% success rate
Kvint et al.	Retrospect	31	15.2	Canine	Permanen	Immature	Maxillary i	Modified A	Adhesive f	Root devel	0.84	4.3	0.097	Canines had good but lower success rate; 3 extractions noted
Meinzer et	Prospectiv	24	14	Canine	Permanen	Mixed	Maxillary a	ATT with s	Splint (wir	Initial heal	1	0.06	0	No-antibiotic group had higher pathology and pain scores; more postoperative complications
Meinzer et	Prospectiv	21	14	Canine	Permanen	Mixed	Maxillary a	ATT with s	None or m	Initial heal	1	0.06	0	Intermediate healing benefit; fewer complications than no-antibiotic group
Meinzer et	Prospectiv	22	14	Canine	Permanen	Mixed	Maxillary a	ATT with s	Splint (wir	Initial heal	1	0.06	0	No added healing benefit over single-shot group; risks of side effects higher
Murata et	Case repor	1	16	Premolar	Permanen	Mature	Maxillary r	Immediate	Suture or s	PDL health	1	1.5	0	Innovative use of dentin cement enhances healing
Nethander	Prospectiv	8		Premolar	Permanen	Immature	Mandibula	Two-stage	Suture or s	Retention	0.714	1	1	Two-stage technique showed 89% retention; all 8 failures occurred in extracted group
Nethander	Prospectiv	67		Premolar	Permanen	Immature	Mandibula	Modified A	Suture onl	Retention	0.985	1	0	Two-stage technique showed 89% retention; all 8 failures occurred in extracted group
Nethander	Prospectiv	57	30.7	Premolar	Permanen	Mature	Mandibula	Delayed A	Not specifi	PDL health	0.77	1	0.105	2-stage healing improves outcomes for mature transplants
Nimčenko	Case series	15	18.1	Molar	Permanen	Immature	Mandibula	Modified A	Splint (wir	PDL health	0.87	0.75	0.133	Third molars viable replacement with close follow-up
Park et al.	Retrospect	47	21	Third mola	Permanen	Mature	Mandibula	Modified A	Suture or s	Retention	0.872	1	0.128	Younger patients had significantly higher survival rates across time points
Park et al.	Retrospect	64	47.5	Third mola	Permanen	Mature	Mandibula	Immediate	Suture or s	Retention	0.875	1	0.125	Despite higher RCT rate, older patients had comparable but not improved outcomes
Suwanapo	Retrospect	50	17.8	Molar and	Permanen	Mixed	Mandibula	Modified A	Suture onl	Clinical an	100	1	0	All teeth survived; pulp healing significantly influenced by age, arch type, root maturity, and extra-alveolar time
Waikakul e	Prospectiv	54		Molar	Permanen	Mixed	Maxillary a	Modified A	Not specifi	PDL health	0.93	1	0.074	Flexible fixation and short extra-alveolar time preserved vitality
