# Supplementary material for: Autogenous Transplantation of Teeth Across Clinical Indications: A Systematic Review and Meta-Analysis
Source: J Clin Med. 2025 Jul 18;14(14):5126. doi: 10.3390/jcm14145126 (PMC12295735; doi:10.3390/jcm14145126)
Supplement: Supplementary file 1 [file jcm-14-05126-s001.zip › Supplementary File S3.pdf]

Study	Study Design	Sample Size (Number of Teeth)	Tooth Type	Tooth Dentition	Root Development Stage	Recipient Sites	Surgical Technique Notes	Success Definition	Survival Rate (%)	Follow-up Duration (years)	Failures (%)	Key Findings
Alkofahi et al., 2020	Prospective	1	Molar	Permanent	Immature	Not reported	Immediate ATT	Root development	100.00%	2	0.00%	PRF may enhance periodontal healing and root development
Cahuana-Bartra et al., 2020	Prospective	1	Premolar	Permanent	Immature	Maxillary central incisor	3D-guided ATT	Clinical and radiographic success	100.00%	1	0.00%	3D printing minimized extraoral time and improved placement accuracy
Cui et al.	Retrospective	20-Sep	Molar	Permanent	Mature	Not reported	Standard ATT	Clinical and radiographic success	100.0%/60.0%	0.7	0.0%/40.0%	RCT improved success
Dixit et al. (2024)	Retrospective	12-Dec	Third molar	Permanent	Mature/Immature	Mandibular second molar	Standard ATT	Unspecified / general success	95.0%/95.0%	0.5	8.3%/8.3%	Similar success in mature & immature
Han et al. (2025)	Retrospective	167	Third molar	Permanent	Mature	Mandibular first or second molar	Standard ATT	Clinical and radiographic success	98.80%	0.5	2.40%	RCT led to better outcomes
Hoss et al. (2021)	Retrospective	53	Anterior (canines and incisors)	Primary	Immature	Primary incisor or premolar	Modified ATT	Root development	77.00%	0.8	58.50%	Useful for space & tissue development

Huth et al. (2013)	Retrospective	57	Canines, molars, premolars, incisors	Permanent	Mixed	Not specified	Standard ATT	Function without pain or pathology	96%	1.6	4.00%	High survival rate; success lower at 74%. Satisfaction high; success associated with oral hygiene and bone healing
Keranmu et al. (2021)	Retrospective	26/26	Third molar	Permanent	Mature	Mandibular premolar	ATT with graft/biologic/3D-guided ATT	Clinical and radiographic success	100.0%/92.3%	0.5	0.0%/7.7%	CGF group had 100% success; faster healing, less pain, better initial stability than control
Kim et al. (2005)	Retrospective	182	Molar and premolar	Permanent	Mixed	Not reported	Standard ATT	PDL health / absence of complications	95.50%	0.7	4.40%	<10 min extraoral time correlated with better prognosis
Kimura et al. (2021)	Retrospective	1	Third molar	Permanent	Mature	Mandibular second molar	Immediate ATT	PDL health / absence of complications	100.00%	29	0.00%	Demonstrated 29-year long-term success of mature ATT

Kvint et al. (2010)	Retrospective	24/71/31	Premolar/third molar/canine	Permanent	Immature/mixed/immature	Maxillary incisor, canine, premolar, and molar	Standard ATT/standard ATT/modified ATT	Root development	100.0%/79.0%/84.0%	4.3	0.0%/21.1%/9.7%	Premolars transplanted to maxillary incisor region had 100% success; most favorable outcome group
Meinzer et al. (2025)	Prospective	24/21/22	Canine	Permanent	Mixed	Maxillary anterior	ATT with storage medium	Initial healing (SSP/SPI)	100.00%	0.1	0.00%	No-antibiotic group had higher pathology and pain scores; more postoperative complications
Murata et al. (2022)	Prospective	1	Premolar	Permanent	Mature	Maxillary molar	Immediate ATT	PDL health / absence of complications	100.00%	1.5	0.00%	Innovative use of dentin cement enhances healing
Nethander (1998)	Retrospective	Aug-67	Premolar	Permanent	Immature	Mandibular premolar	Two-stage ATT/modified ATT	Retention without pathology	71.4%/98.5%	1	100.0%/0.0%	Two-stage technique showed 89% retention; all 8 failures occurred in extracted group

Nethander et al. (1988)	Prospective	57	Premolar	Permanent	Mature	Mandibular molar	Delayed ATT	PDL health / absence of complications	77.00%	1	10.50%	2-stage healing improves outcomes for mature transplants
Nimčenko et al. (2014)	Retrospective	15	Molar	Permanent	Immature	Mandibular second molar	Modified ATT	PDL health / absence of complications	87.00%	0.8	13.30%	Third molars viable replacement with close follow-up
Park et al. (2022)	Retrospective	47/64	Third molar	Permanent	Mature	Mandibular first molar	Modified ATT/Immediate ATT	Retention without pathology	87.2%/87.5%	1	12.8%/12.5%	Younger patients had significantly higher survival rates across time points
Pongrel (1987)	Retrospective	416	Maxillary canines, third molars, lower premolars	Permanent	Mature	First molars, central incisors, premolars	Standard	No signs of resorption, ankylosis, or pathology; stable and healthy gingiva	72.00%	2	28.00%	Higher success (94%) when atraumatic technique and <3 min extraoral time used

Suwanapong et al. (2021)	Retrospective	50	Molar and premolar	Permanent	Mixed	Mandibular or maxillary molar	Modified ATT	Clinical and radiographic success	100%	1	0.00%	All teeth survived; pulp healing significantly influenced by age, arch type, root maturity, and extra-alveolar time
Waikakul et al. (2011)		54	Molar	Permanent	Mixed	Maxillary anterior or mandibular posterior	Modified ATT	PDL health / absence of complications	93.00%	1	7.40%	Flexible fixation and short extra-alveolar time preserved vitality
