# Supplementary material for: Autogenous Transplantation of Teeth Across Clinical Indications: A Systematic Review and Meta-Analysis
Source: J Clin Med. 2025 Jul 18;14(14):5126. doi: 10.3390/jcm14145126 (PMC12295735; doi:10.3390/jcm14145126)
Supplement: Supplementary file 1 [file jcm-14-05126-s001.zip › Supplementary File S1.pdf]

```
# Supplementary File 1: R_code_ATT_MetaAnalysis.R
# Title: Meta-Analysis of Autogenous Tooth Transplantation Success Rates
# Authors: Martin Baxmann, Karin Christine Huth, Krisztina Kárpáti, and Zoltán Baráth
# Date: June, 2025
# Description: R script used for quantitative synthesis in the systematic review and meta-analysis
# "Autogenous Transplantation of Teeth Across Clinical Indications"

# 1. Load necessary packages
library(metafor)
library(meta)
library(readxl)
library(dplyr)

# 2. Read in data
# Replace 'your_data.xlsx' and 'Sheet1' with your actual file and sheet name
data <- read_excel("your_data.xlsx", sheet = "Sheet1")

# 3. Calculate effect sizes using logit-transformed proportions
# Assumes columns: events = number of successes, n = total number of transplants
dat <- escalc(measure = "PLO", xi = events, ni = n, data = data, slab = paste(Author, Year))

# 4. Run random-effects meta-analysis model
res <- rma(yi, vi, data = dat, method = "REML")
summary(res)

# 5. Forest plot
forest(res, xlab = "Logit-transformed Success Proportion",
    atransf = transf.ilogit,
    at = transf.logit(c(0.6, 0.8, 0.9, 0.95, 0.98, 1.0)),
```

```
    digits = 2)

# 6. Funnel plot to assess publication bias
funnel(res, xlab = "Logit-transformed Effect Size", ylab = "Standard Error")
regtest(res, model = "lm")
```
